# Influence of a Structured Microbiological Endotracheal Monitoring Program on the Outcome of Critically Ill COVID-19 Patients: An Observational Study

**DOI:** 10.3390/jcm12175622

**Published:** 2023-08-28

**Authors:** Miriam Dibos, Stefanie Julia Haschka, Rami Abbassi, Jochen Schneider, Roland M. Schmid, Sebastian Rasch, Tobias Lahmer

**Affiliations:** Department of Internal Medicine II, School of Medicine, University Hospital Rechts der Isar, Technical University of Munich, Ismaninger Str. 22, 81675 Munich, Germany; miriam.dibos@mri.tum.de (M.D.);

**Keywords:** COVID-19, SARS-CoV-2, bacterial spectrum, ventilator-associated pneumonia, ICU

## Abstract

Background: In past influenza pandemics and the current COVID-19 pandemic, bacterial endotracheal superinfections are a well-known risk factor for higher morbidity and mortality. The goal of this study was to investigate the influence of a structured, objective, microbiological monitoring program on the prognosis of COVID-19 patients with mechanical ventilation. Methods: A structured microbiological monitoring program (at intubation, then every 3 days) included collection of endotracheal material. Data analysis focused on the spectrum of bacterial pathogens, mortality, as well as intensive care unit (ICU), hospital, and mechanical ventilation duration. Results: A total of 29% of the patients showed bacterial coinfection at the time of intubation, and within 48 h, 56% developed ventilator-associated pneumonia (VAP). Even though patients with VAP had significantly longer ICU, hospital, and mechanical ventilation durations, there was no significant difference in mortality between patients with VAP pneumonia and patients without bacterial infection. Conclusion: VAP is a common complication in COVID-19 patients. In contrast to already published studies, in our study implementing a structured microbiological monitoring program, COVID-19 patients with bacterial coinfection or VAP did not show higher mortality. Thus, a standardized, objective, microbiological screening can help detect coinfection and ventilator-associated infections, refining anti-infective therapy and positively influencing patient outcomes.

## 1. Introduction

Bacterial ventilator-associated pneumonia (VAP) is one of the most common nosocomial infectious complications of mechanical ventilation. As such, VAP leads to longer duration of mechanical ventilation, longer intensive care unit (ICU) stay, and higher mean hospital charges [1,2]. Furthermore, VAP complicates the outcome of critically ill patients [2].

Unlike COVID-19, bacterial VAP in patients with influenza has been thoroughly studied. In past influenza pandemics, bacterial VAP increased the risk for severe illness and higher mortality [3,4,5,6]. In patients with influenza requiring mechanical ventilation, the main pathogens causing secondary superinfections are *Streptococcus pneumoniae* (*S. pneumoniae*), *Streptococcus pyogenes* (*S. pyogenes*), *Staphylococcus aureus* (*S. aureus*), and *Haemophilus influenzae* (*H. influenzae*) [7,8]. To understand the pathophysiological mechanisms, animal studies revealed that the initial viral infection led to damaged bronchoalveolar physical barriers with permissive lung epithelial surfaces, which makes bacterial attachment easier [9,10]. 

Similar mechanisms are suspected in critically ill COVID-19 patients. Previous studies investigated the prevalence of coinfection of SARS-CoV-2 patients and influenza patients upon hospital admission. Delhommeau et al. showed that bacterial coinfection in SARS-CoV-2 patients was less frequent than in patients with influenza (8.2% for SARS-CoV-2 versus 24.8% for influenza) [11]. Furthermore, the prevalence of coinfection was not associated with a higher mortality or with the development of VAP [11]. Concerning VAP alone, the overall incidence of VAPs in Europe has been declining over the past decade. This is hypothesized to be due to an increased awareness of various preventing strategies due to recently published guidelines for the management of VAP [2,12]. Nonetheless, in regard to COVID-19 patients, previous studies showed that these patients are prone to a high prevalence of bacterial VAP and invasive aspergillosis complicating their clinical course [4,12,13,14]. Especially due to the long duration of mechanical ventilation in COVID-19 patients, they are under an increased risk of VAP [6,13]. Therefore, regular microbiological testing for VAP could be essential especially in COVID-19 patients in order to quickly adjust antibiotic treatment to reduce mortality. Furthermore, to avoid facilitating the growth of multidrug-resistant bacteria, antimicrobial stewardship programs are critical to reduce the broad use of empirical antibiotics [4,12,13,14]. 

Clinical differentiation between respiratory deterioration caused by COVID-19 or VAP is difficult, and clinical deterioration is a subjective criterion. Therefore, to avoid a VAP remaining undetected, a regular microbiological testing program and an according test-appropriate adjustment of antibiotic therapy may be crucial. Hence, we recognized the need for an objective and structured microbiological monitoring program. To further assess this, we conducted a single-center observational study. The primary objective was to identify characteristics of patients with coinfection and VAP and the spectrum of bacterial pathogens in coinfection and VAP. The secondary objective was to analyze the influence of a structured, microbiological monitoring program on the prognosis of intubated critically ill COVID-19 patients. The choice and duration of antibiotic therapy and potential benefits of the objective microbiological screening on antibiotic therapy is part of another ongoing study.

## 2. Materials and Methods

### 2.1. Study Population

This observational single-center study analyzed 241 critically ill patients with confirmed SARS-CoV-2 infection who were hospitalized between 1 March 2020, and 27 April 2022, at University Hospital Rechts der Isar of the Technical University of Munich. Of these 241 patients, only ICU patients with severe or critical COVID-19 (SpO2 < 94% on room air, respiratory rate > 30/min or lung infiltrates > 50%) who required mechanical ventilation were analyzed and eligible for study inclusion. After applying these criteria, 169 patients with severe COVID-19 pneumonia were included into the study. According to general acute respiratory distress syndrome (ARDS) standards, percutaneous tracheostomy was performed in patients with prolonged mechanical ventilation.

### 2.2. Microbiological Screening

**Figure 1:**
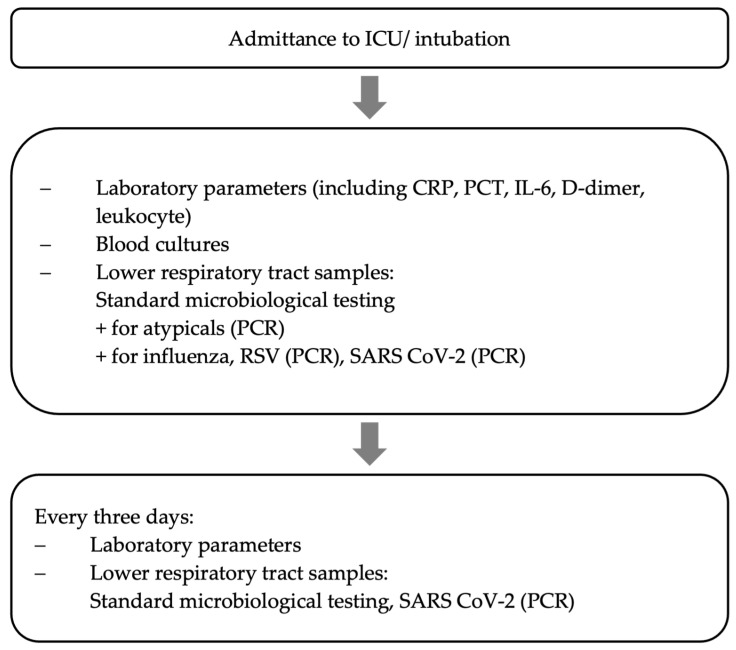
Data collection [15].

All critically ill patients with severe COVID-19 pneumonia were screened microbiologically throughout the time of mechanical ventilation using a standardized study protocol, which is already well-established and published by our group [15] (see Figure 1). This structured microbiological monitoring program (immediately after intubation, then every three days) included quantitative cultures and a PCR panel of bronchoalveolar lavage (BAL) fluid samples or non-directed bronchial lavage (see Figure 1). 

Either BAL or a non-directed bronchial lavage (NBL) using a closed system was used to obtain material. Especially during the early months of the pandemic, the reduced risk of aerosolization when performing NBL instead of BAL was beneficial. Diagnostic accuracy identifying VAP has been proven similar between BAL and NBL [16]. In cases of clinical deterioration, endobronchial sample collection was moved ahead in time. 

Applying the protocol explained above, the following definitions were used to differentiate between bacterial coinfection and VAP. Bacterial infection at time of intubation or within 48 h of intubation in respiratory secretions (either BAL or NBL) was counted as bacterial coinfection. Although this is a well-established definition used in the literature, differentiating between bacterial coinfection and colonialization is not always possible. Nonetheless, both bacterial coinfections and colonialization at time of intubation have been shown to be associated with a higher mortality depending on the documented pathogen and the number of comorbidities [11]. In regard to VAP, we counted bacterial superinfection more than 48 h after intubation as VAP according to the literature [17,18]. Furthermore, to assess potential risk factors for the development of VAP, the baseline characteristics of patients who did and did not acquire VAP throughout their ICU stay were compared with each other. 

Following current institutional and national guidelines, all patients received the same standard of care procedure during the SARS-CoV-2 infection [19]. Depending on the stage and severity of COVID-19, some patients were additionally treated with dexamethasone, monoclonal antibodies, or tocilizumab. Depending on the clinical course and inflammation markers, blood cultures underwent microbiological testing. 

### 2.3. Data and Statistical Analysis

For data collection, we used the database of our hospital information system (SAP Clinical Data Warehouse Cloud ©). Statistical analysis was performed using Prism 9, Version 9.4.1 (458), 18 July 2022. Samples were tested for normal distribution using the Shapiro–Wilk test. Normally distributed parameters are presented as mean ± standard deviation and, accordingly, descriptive data without normal distribution as median and interquartile range (IQR). For the analysis of quantitative variables, the t-test and the Mann–Whitney U test were employed. All statistical tests were two-sided with a level of significance (*p*-value) of 5%. 

## 3. Results

### 3.1. Baseline Characteristics of Patients with Coinfection and VAP

**Figure 2:**
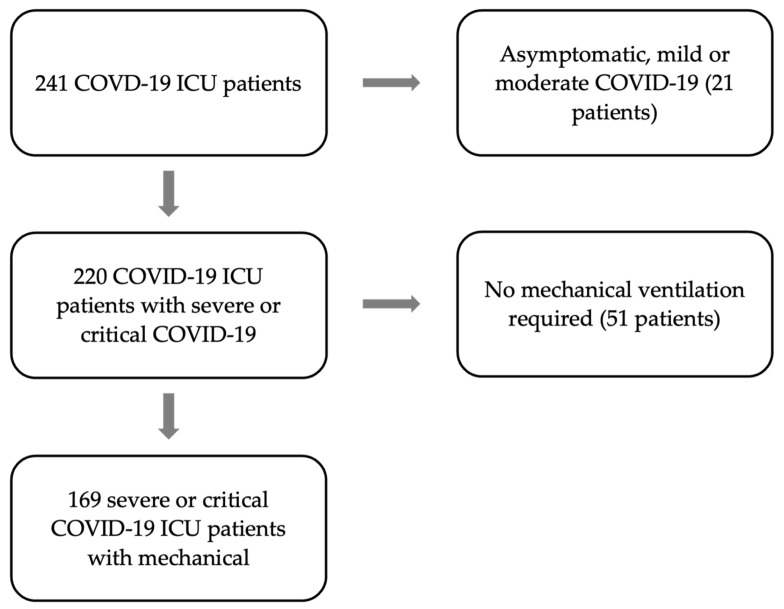
Flow chart of the enrolled patients between March 2020 to April 2022, arrows pointing to the right indicate exclusion and down-pointing arrows indicate inclusion.

During the study period, 241 COVID-19 patients were treated in our ICU. Out of the 241 screened patients, patients were excluded because of a mild or asymptomatic infection (*n* = 21) or because no mechanical ventilation was required (*n* = 51) (Figure 2). The remaining 169 patients with severe or critical COVID-19 requiring mechanical ventilation were investigated further.

The baseline characteristics of the remaining 169 patients are summarized in Table 1. A total of 29% of the patients showed bacterial coinfection at the time of intubation or within 48 h (*n* = 49). At least one episode of VAP could be detected in 56.2% of the patients (*n* = 95). A total of 26.6% of the patients did not have coinfection and did not develop bacterial infection throughout the course of mechanical ventilation (*n* = 45). 

No difference in age or gender could be identified between patients with coinfection, with or without VAP. 

COVID-19 specific therapy with corticosteroids was associated with a higher risk of VAP development (*p* < 0.001, see Table 1). Patients with VAP spent significantly more time on the ICU (23 days, IQR 23 vs. 16 days, IQR 18, *p* < 0.001), on mechanical ventilation (19 days, IQR 21.5 vs. 10 days, IQR 14, *p* < 0.001), and in the hospital (33 days, IQR 22.5 vs. 20 days, IQR 23, *p* = 0.002) (see Table 1). Nonetheless, there was no significant difference between the outcome of patients with and without VAP (37.9% vs. 48.9%, *p*-value 0.271).

Of the 49 patients with coinfection, 20 additionally developed at least one episode of VAP. Patients with coinfection and additional VAP had significantly longer ICU, hospital duration, and days on ventilator compared to patients with coinfection alone. The outcome of patients with coinfection and patients with coinfection and additional development of VAP however did not differ.

### 3.2. Etiology and Incidence of Coinfection and VAP

The etiology of coinfection and of VAP and its incidence are demonstrated in Table 2. In coinfection as well as in VAP, the incidence of Gram-negative bacteria was higher than the incidence of Gram-positive bacteria (66% infections with Gram-negative bacteria in coinfection vs. 81.5% in VAP). In coinfection, the main etiological Gram-negative agents were *Morganella* species (14.3% of all confections) and *Escherichia* species (13.2% of all confections). In VAP, the main etiological Gram-negative agents were *Klebsiella* species (25%). Both in coinfection and in VAP, *S. aureus* was the main Gram-positive bacterium (31.9% in coinfection vs. 18.6% in VAP).

Comparing inflammation markers (C reactive protein (CRP), procalcitonin (PCT), leukocyte count, and interleukin 6 (IL-6)) at the time of intubation between patients with vs. without later development of VAP, there was a significant difference of CRP and PCT in patients with vs. without VAP (median CRP 12 mg/dl vs. 18 mg/dl, *p*-value 0.042, median PCT 0.4 ng/mL vs. 0.8 ng/mL, *p*-value 0.041). Laboratory parameters are listed in Table S1, Supplementary Material.

### 3.3. Bacterial Coinfection vs. Ventilator-Associated Pneumonia

**Figure 3:**
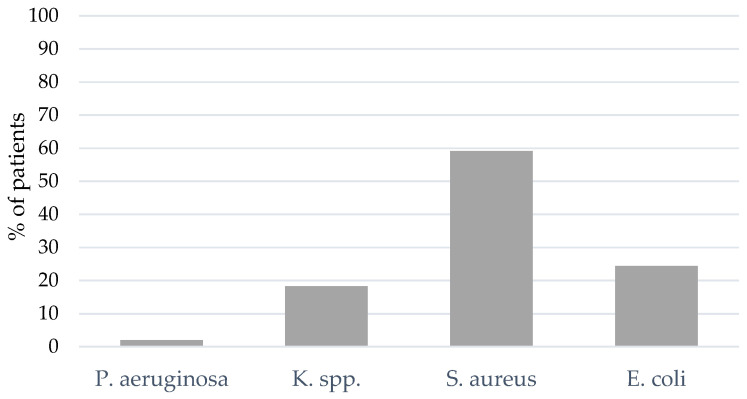
BAL and NBL results for bacterial pathogens of coinfection. *Klebsiella* species (K. spp.).

A total of 29% of the patients presented with bacterial coinfection at the time of intubation or within 48 h (*n* = 49). Only the most frequent bacterial pathogens are presented in Figure 3 below. The most frequent bacterial pathogen was *S. aureus* (59.2% of the patients), followed by *Escherichia coli* (*E. coli*, 24.5%) and *Klebsiella* species (K. spp., 18.4%) (see Figure 3). Figure S1 in the Supplementary Materials shows the bacterial spectrum of pathogens identified within 48 h after intubation in percentages representing the portion of all pathogens found.

**Figure 4:**
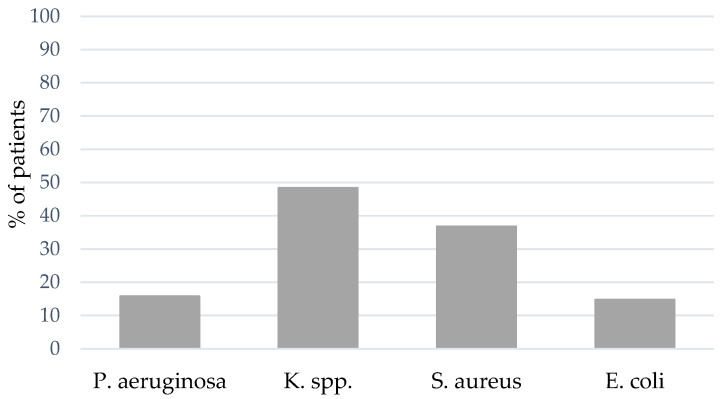
BAL and NBL results for bacterial pathogens of VAP.

At least one episode of VAP (either with *S. aureus*, K. spp., *Pseudomonas aeruginosa* (*P. aeruginosa*), *E. coli* or others) could be detected in 56.2% of the patients (*n* = 95). The spectrum of bacterial pathogens differed between coinfection and VAP: The portion of coinfections caused by Gram-positive bacterial pathogens was larger, whereas an increasing portion of Gram-negative bacterial pathogens were causal for VAP. In 48.8% of the patients with VAP, K. spp. was the bacterial pathogen causing VAP, followed by *S. aureus* (36.8%) and an increasing percentage of *P. aeruginosa* (15.8%) compared to coinfection (see Figure 4). The bacterial spectrum of VAP is presented in Figure S2 in Supplementary Materials.

**Figure 5:**
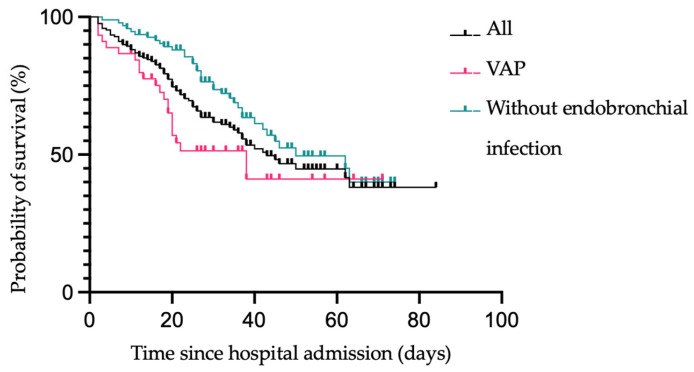
Kaplan–Meier survival estimates of all patients vs. patients without endobronchial infection vs. patients with VAP following hospital admission.

Survival estimates of patients with VAP, without endobronchial infection, and of all patients are shown in Figure 5. No significant difference between the outcome of patients with VAP or without any bacterial endotracheal infection could be found (see Figure 5).

### 3.4. S. aureus Coinfection and Ventilator-Associated Pneumonia

**Figure 6:**
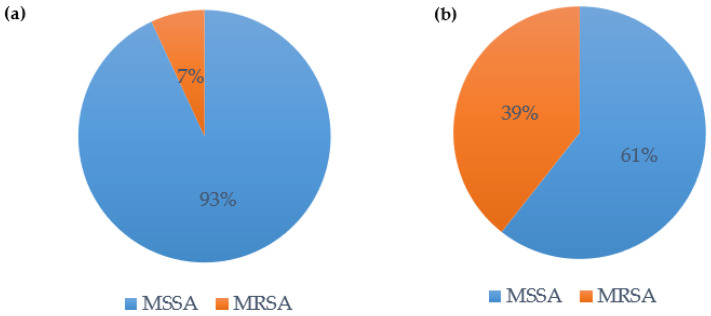
(**a**) Bacterial coinfection with *S. aureus*—distribution MSSA vs. MRSA; (**b**) VAP with *S. aureus*—distribution MSSA vs. MRSA.

In our cohort, *S. aureus* was the most frequent pathogen of bacterial coinfection: 59.2% of the patients with bacterial coinfection were coinfected with *S. aureus* (*n* = 29). In these patients, methicillin-sensitive *S. aureus* (MSSA) was the more prevalent *S. aureus* (93.1%, see Figure 6a). In patients with *S. aureus*-induced VAP, the distribution changed: MSSA only caused 60.6% of the superinfections, whereas methicillin-resistant *S. aureus* (MRSA) caused 39.4% of the superinfections (see Figure 6b).

Comparing the general criteria of patients with and without *S. aureus* induced confection or VAP, we could show that patients with *S. aureus* required longer mechanical ventilation (22 days, IQR 27.5 vs. 16, IQR 18, *p*-value 0.001) and a longer ICU stay (15.5 days, IQR 26.5 vs. 12 days, IQR 16, *p*-value 0.025, see Table 3). No significant difference between the outcome could be detected (mortality of 38.3% vs. 46.8%, *p*-value 0.333).

We compared infection parameters of all patients at time of intubation (see Table S2 Supplementary Material). Median CRP, PCT, leucocyte count, and IL-6 were not different in patients with or without later *S. aureus* infection (see Table S2, Supplementary Material).

## 4. Discussion

To the best of our knowledge, this is the first study that reports a structured microbiological screening in critically ill COVID-19 patients that includes the analysis of the bacterial spectrum of coinfection and VAP at predefined intervals and its influence on the outcome of affected patients. 

Analyzing the bacterial spectrum of pulmonal coinfection in COVID-19 patients, *S. aureus* and *E. coli* were the most common pathogens causing coinfections in our cohort. This is in line with other studies such as by Russel et al. and Pickens et al. who found *S. aureus*, *H. influenzae* and *S. aureus*, and *Streptococcus* species as the most prevalent pathogens [4,20]. The spectrum of pathogens causing VAP in our study group is coherent with what has been published for COVID-19 patients and for patients with acute respiratory distress syndrome and VAP in general. Especially Gram-negative bacteria such as *P. aeruginosa*, *Enterobacteriaceae* species, *K. pneumoniae*, and MRSA are most common [4,20,21]. The high incidence of *P. aeruginosa* and MRSA might lead to a frequent use of empirical antibiotics, therefore encouraging the development of multidrug-resistant bacteria. As reported in other studies, treatment with immunosuppressants such as dexamethasone or other corticosteroids as recommended by the COVID-19 S3 guidelines, also increases the risk of secondary bacterial pulmonary infections in our study [19,22]. An ongoing study by our group investigates the benefits of this objective microbiological screening on the choice and duration of antibiotic therapy. In this ongoing study, infection parameters, clinical parameters, and microbiological findings are interpreted to help adjust the clinician’s decision to escalate or deescalate antibiotic therapy.

In our study, we found a prevalence of bacterial coinfection at time of intubation of 29%, which is higher than what has been previously published in studies analyzing critically ill COVID-19 patients. Pickens et al. reported a coinfection rate of 21%, whereas Rouzé et al. reported a coinfection rate of 9.7% [4,23]. Comparing rates of VAP between COVID-19 and severe influenza cases, our study and others found a generally higher prevalence of VAP in COVID-19 patients [6,13,24,25,26]. In our study, 56.2% developed VAP, whereas VAP prevalence in COVID-19 patients in the literature is reported to be between 33% to 64% [6,12,26,27]. One reason for the different prevalence of bacterial coinfections and VAP between our study and others may be the method and timing of material collection. In previous studies, most samples were tracheal aspirates, whereas we required BAL or NBL in our study which have higher sensitivity and specificity [23]. The timepoints of collection of microbiological material vary from study to study but are in most cases only based on clinical suspicion and potential deterioration (e.g., laboratory findings, clinical, or radiological signs of infection) [4]. In a recent study by Pickens et al., BAL was collected routinely after intubation. Follow-up testing was based on clinical suspicion assessing bacterial coinfection or VAP [4]. This leads to a significant delay in diagnosis and adjustment of treatment. Another contributing factor for the different prevalence of coinfection and VAP in our study and others might be patient selection, as we only included patients with critical COVID-19 and patients requiring mechanical ventilation. Several additional reasons for the generally increased prevalence of VAP in COVID-19 patients between our study and others could be considered: therapy-associated immune modulation (corticosteroids, monoclonal antibodies, see Table 1), the high dosage of sedation, frequent prone-positioning, and prolonged duration of mechanical ventilation in COVID-19 patients are suspected contributors [28,29,30,31]. 

Comparing median ventilation duration, ICU days, and hospital duration between patients with and without VAP, we show that COVID-19 patients with VAP required significantly longer mechanical ventilation, as well as longer ICU and hospital stays, leading to higher morbidity rates and treatment costs. Our data are consistent with other studies investigating ventilation, ICU, and hospital durations of COVID-19 patients with VAP [30,32]. Although these studies showed the association of a higher 28-day mortality in SARS-CoV2 patients with VAP, others did not find increased mortality rates [12,30,32]. Our study with a closer microbiological monitoring confirms the similar mortality of bacterial coinfections and VAP, although VAP in ARDS is normally associated with a worse outcome. Thus, an objective microbiological screening method could lead to faster, better adjusted anti-infective therapy, differentiating between the normal course of COVID-19 pneumonia and VAP and, therefore, might reduce mortality rates.

We show that infection parameters cannot predict later development of VAP (see Table S2, Supplementary Material). A major strength of our study is the routine regular microbiological testing over a timespan of more than two years during which we continuously collected data. Based on our study on the reviewed cases, we therefore recommend considering a closer, objective microbiological monitoring in all patients with ARDS with potential development of VAP. This might help adjust antibiotic therapy more quickly in patients with COVID-19 but also in patients with different etiologies of respiratory deterioration. Despite the global decline in COVID-19 infections due to vaccination programs, an objective microbiological testing might still have consequences on treatment of mechanically ventilated patients with VAP.

Our study has several limitations. First, it is a single-center observational study with a standardized screening tool without a control group having received no screening. 

Second, although we and others have already shown that NBL (instead of BAL) is a comparable method to obtain bronchial lavage material in mechanically ventilated patients, it still is a relatively new alternative to BAL, which needs further investigation [15,16,33]. Nonetheless, NBL has proven to be a safe method with a similar diagnostic accuracy diagnosing VAP as BAL [16]. Third, although the standardized screening for new bacterial pathogens and new episodes of VAP enables the quick adjustment of anti-infective therapy, the potential overdiagnosis and overtreatment of VAP must be considered. 

## 5. Conclusions

In summary, bacterial coinfection and ventilator-associated pneumonias are very common complications of critically ill COVID-19 patients with mechanical ventilation. As infection parameters and clinical suspicion do not work as objective predictors for the development of VAP, our data indicate the importance of a standardized screening method, especially as screening and quick identification of bacterial pathogens causing VAP facilitate a timely adjustment of therapy. A microbiological screening based only on clinical suspicion might increase the risk of delayed diagnosis of a new bacterial pathogen causing VAP and, following this, delayed adjustment of anti-infective therapy. This can help develop antimicrobial stewardship programs to reduce the increasing percentage of multidrug-resistant bacteria. Therefore, a standardized and objective microbiological screening as presented in our study can help to quickly identify and treat bacterial superinfection, which might be beneficial on morbidity and mortality rates.

## Figures and Tables

**Table 1:** Comparison of patients with coinfection, with VAP and without endobronchial infection. *p*-value comparing patients with VAP with patients without endobronchial infection.

Patients	Total (169)	Coinfection (49)	VAP (95)	No Infection (45)	*p*-Value
Median age—years (IQR)	63 (23)	63 (22)	63 (24)	63 (19)	0.633
Male sex—% (*n*)	74 (125)	77.6 (38)	76.8 (73)	62.2 (28)	0.105
COVID-specific therapy—% (*n*)					
- Remdesivir	11.8 (20)	2 (1)	15.8 (15)	11.1 (5)	0.607
- Tocilizumab	7.1 (12)	14.3 (7)	7.4 (7)	2.2 (1)	0.278
- Monoclonal antibodies	14.8 (25)	20.4 (10)	11.6 (11)	15.6 (7)	0.591
- Corticosteroids	81.1 (137)	87.8 (43)	88.4 (84)	62.2 (28)	<0.001 (*)
ECMO—% (*n*)	10.1 (17)	8.2 (4)	12.6 (12)	8.9 (4)	0.584
Median duration of ICU stay (IQR)	18 (21)	17 (22)	23 (23)	13 (11)	<0.001 (*)
Median duration of hospital stay (IQR)	26 (22)	25 (28)	33 (22.5)	20 (23)	0.002 (*)
Median number of ventilator days (IQR)	15 (19)	14 (18)	19 (21.5)	10 (14)	<0.001 (*)
Outcome					
Death—% (no.)	43.8 (74)	55.1 (27)	37.9 (36)	48.9 (22)	0.271

Abbreviations: IQR: interquartile range, ECMO: extracorporal membrane oxygenation, * indicating statistical significance.

**Table 2:** All bacterial pathogens detected in coinfections and in VAP. Percent of all pathogens.

	Coinfection	VAP
Gram-negative bacteria		
*Pseudomonas aeruginosa*	1.1	8
*Stenotrophomonas maltophilia*	1.1	2.7
*Acinetobacter* spp. (Acinetobacter pittii/dijkshoorniae + *Acinetobacter baumanii*)	2.2	5.9
*Brevundimonas* species (spp.)	0	0.5
*Enterobacteriaceae*		
*Escherichia* spp.	13.2	8
*Citrobacter* spp.	5.5	4.8
*Enterobacter* spp.	4.4	6.4
*Hafnia* spp.	1.1	0
*Morganella* spp.	14.3	8.5
*Proteus* spp.	3.3	4.3
*Serratia* spp.	5.5	5.3
*Klebsiella* spp.	9.9	25
*Haemophilus influenzae*	3.3	2.1
*Moraxella catarrhalis*	1.1	0
Gram-positive bacteria		
*Staphylococcus aureus*	31.9	18.6
*Streptococcus agalactiae*	2.2	0

**Table 3:** Comparison of patients with *S. aureus* and without *S. aureus* infection.

	Total (169)	+ *S. aureus* (60)	− *S. aureus* (109)	*p*-Value
Median age—years (IQR)	63 (23)	61 (20.3)	63 (24)	0.706
Male sex—% (*n*)	74 (125)	78.3 (47)	71.6 (78)	0.366
COVID-specific therapy—% (*n*)				
- Remdesivir	11.8 (20)	8.3 (5)	13.8 (15)	0.333
- Tocilizumab	7.1 (12)	8.3 (5)	6.4 (7)	0.756
- Monoclonal antibodies	14.8 (25)	16.7 (10)	13.8 (15)	0.654
- Corticosteroids	81.1 (137)	88.3 (53)	77.1 (84)	0.1
ECMO—% (*n*)	10.1 (17)	8.3 (5)	11 (12)	0.61
Median duration of ICU stay (IQR)	18 (21)	22 (27.5)	16 (18)	0.001 (*)
Median duration of hospital stay (IQR)	26 (22)	27 (29.3)	26 (21)	0.171
Median number of ventilator days (IQR)	15 (19)	15.5 (26.5)	12 (16)	0.025 (*)
Outcome				
Death—% (no.)	43.8 (74)	38.3 (23)	46.8 (51)	0.333

Abbreviations: * indicating statistical significance.

## Data Availability

No new data were created or analyzed in this study. Data sharing is not applicable to this article.

## Supplementary Materials

The following supporting information can be downloaded at: https://www.mdpi.com/article/10.3390/jcm12175622/s1, Table S1: Infection parameters at time of intubation of patients with coinfection, VAP, without infection; Table S2: Infection parameters at time of intubation of patients with *S. aureus* infection, without *S. aureus* infection, without infection; Figure S1: Bacterial spectrum of coinfection, where 100% represents all identified bacterial pathogens. Figure S2: Bacterial spectrum of VAP, where 100% represents all identified bacterial pathogens.
