# Supplementary material for: Influence of a Structured Microbiological Endotracheal Monitoring Program on the Outcome of Critically Ill COVID-19 Patients: An Observational Study
Source: J Clin Med. 2023 Aug 28;12(17):5622. doi: 10.3390/jcm12175622 (PMC10488947; doi:10.3390/jcm12175622)
Supplement: Supplementary file 1 [file jcm-12-05622-s001.zip › Supplementary materials.pdf]

Supplementary files

Table S1: Infection parameters at time of intubation of patients with coinfection, VAP, without infection.

	**Total (169)**	**Coinfection (49)**	**VAP (95)**	**No infection (45)**	**p-value**
Median CRP in mg/dl (IQR)	12.8 (13.3) n=153	10.5 (11.7) n=48	12 (13.3) n=86	18 (12.9) n=38	**0.042 (*)**
Median PCT in ng/ml (IQR)	0.5 (1) n=131	0.5 (1) n=38	0.4 (0.7) n=74	0.8 (1.7) n=36	**0.041 (*)**
Median leucocytes in G/l (IQR)	9.2 (6.2) n=157	9.8 (6.5) n=49	9.4 (5.8) n=88	9.2 (6.5) n=40	0.275
Median Interleukin-6 in pg/ml (IQR)	124 (252.2) n=107	129 (401) n=33	98.5 (221.3) n= 59	183 (235) n=29	0.16

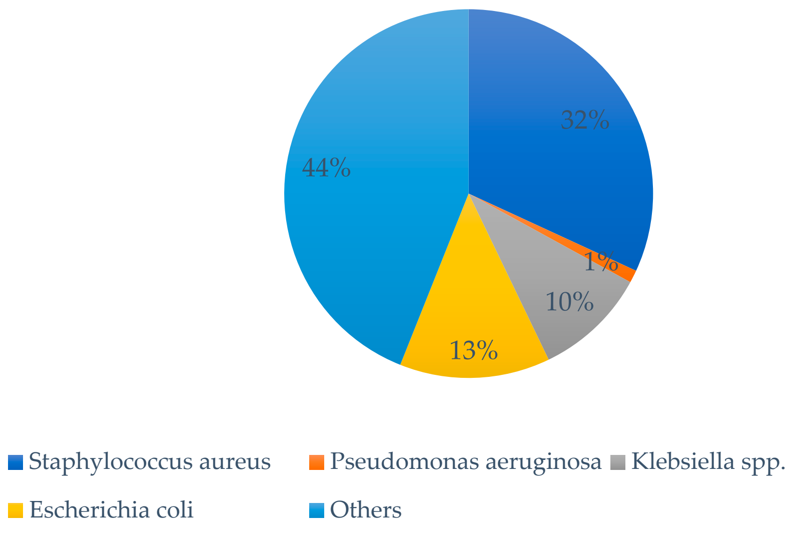

Figure S1: Bacterial spectrum of coinfection, where 100% represent all identified bacterial pathogens

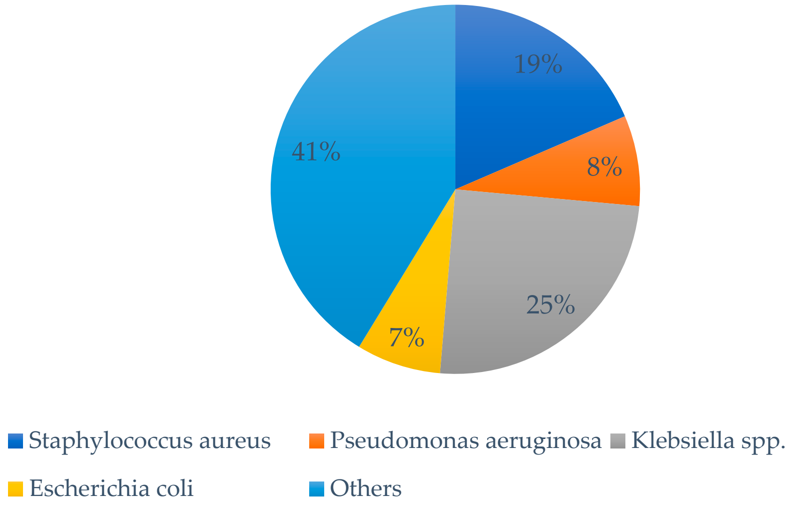

Figure S2: Bacterial spectrum of VAP, where 100% represent all identified bacterial pathogens.

Table S2: Infection parameters at time of intubation of patients with S. aureus infection, without S. aureus infection, without infection

	**Total (169)**	**+ S. aureus infection (60)**	**- S. aureus infection (109)**	**p-value**
Median CRP in mg/dl (IQR)	12.8 (13.3) n=153	11.1 (12.5) n=56	13.4 (13.7) n=97	0.381
Median PCT in ng/ml (IQR)	0.5 (1) n= 131	0.25 (0.6) n=44	0.6 (1.25) n=87	0.065
Median leucocytes in G/l (IQR)	9.2 (6.2) n=157	9.8 (6.2) n=56	8.8 (5.5) n=101	0.221
Median Interleukin-6 in pg/ml (IQR)	124 (252.2) n=107	96.2 (226.8) n=38	146 (258.8) n=69	0.375
